# Atomic structures determined from digitally defined nanocrystalline regions

**DOI:** 10.1107/S2052252520004030

**Published:** 2020-04-10

**Authors:** Marcus Gallagher-Jones, Karen C. Bustillo, Colin Ophus, Logan S. Richards, Jim Ciston, Sangho Lee, Andrew M. Minor, Jose A. Rodriguez

**Affiliations:** a Department of Chemistry and Biochemistry, University of California, Los Angeles (UCLA), Los Angeles, CA 90095, USA; b UCLA-DOE Institute for Genomics and Proteomics, University of California, Los Angeles (UCLA), Los Angeles, CA 90095, USA; c STROBE, NSF Science and Technology Center, University of California, Los Angeles (UCLA), Los Angeles, CA 90095, USA; d National Center for Electron Microscopy, Molecular Foundry, Lawrence Berkeley National Laboratory, California, USA; e Department of Biological Sciences, Sungkyunkwan University, Suwon 16419, Republic of Korea; f Department of Materials Science and Engineering, University of California, Berkeley, California, USA

**Keywords:** electron crystallography, electron-diffraction tomography, nanocrystallography, structure determination, atomic resolution, tilt series

## Abstract

Nanocrystallography has transformed our ability to interrogate the atomic structures of proteins, peptides, organic molecules and materials. By probing atomic level details in ordered sub-10 nm regions of nanocrystals, scanning nanobeam electron diffraction extends the reach of nanocrystallography and in principle obviates the need for diffraction from large portions of one or more crystals. Scanning nanobeam electron diffraction is now applied to determine atomic structures from digitally defined regions of beam-sensitive peptide nanocrystals. Using a direct electron detector, thousands of sparse diffraction patterns over multiple orientations of a given crystal are recorded. Each pattern is assigned to a specific location on a single nanocrystal with axial, lateral and angular coordinates. This approach yields a collection of patterns that represent a tilt series across an angular wedge of reciprocal space: a scanning nanobeam diffraction tomogram. Using this diffraction tomogram, intensities can be digitally extracted from any desired region of a scan in real or diffraction space, exclusive of all other scanned points. Intensities from multiple regions of a crystal or from multiple crystals can be merged to increase data completeness and mitigate missing wedges. It is demonstrated that merged intensities from digitally defined regions of two crystals of a segment from the OsPYL/RCAR5 protein produce fragment-based *ab initio* solutions that can be refined to atomic resolution, analogous to structures determined by selected-area electron diffraction. In allowing atomic structures to now be determined from digitally outlined regions of a nanocrystal, scanning nanobeam diffraction tomography breaks new ground in nanocrystallography.

## Abstract 2

This article demonstrates how a nano-focused electron beam can be used to collect sparse diffraction patterns from ∼40 nm regions of a macromolecular crystal that are subsequently digitally recombined into a tilt series suitable for *ab initio* phasing. By synthesizing signals from specific sub-regions of a crystal after data are collected, the findings provide the basis for capturing atomic level information from mosaic or partially ordered crystals that might otherwise be overlooked.

## 1. Introduction   

A prominent bottleneck to the determination of atomic molecular structures is their formation of well ordered single crystals of a suitable size. As a crystal grows, so too does its likelihood of being disordered (Malkin *et al.*, 1996 ▸). Structural irregularities in a crystal can result in a loss of diffracting power, challenges in data reduction and ultimately increases the difficulty of structure determination (Nave, 1998 ▸). Microfocused X-ray beams overcome some of these challenges, reducing the lower-size limits of crystals from hundreds of micrometres to below ten micrometres (Smith *et al.*, 2012 ▸). Serial crystallography at both synchrotron (Nogly *et al.*, 2015 ▸) and X-ray free-electron laser sources (Schlichting, 2015 ▸) has further reduced crystal-size limits to the sub-micrometre scale at the cost of requiring large numbers of crystals. The recent renaissance in electron diffraction also allows the study of three-dimensional microcrystals (MicroED or cRED) (Shi *et al.*, 2013 ▸; Nannenga *et al.*, 2014 ▸; Cichocka *et al.*, 2018 ▸) and the determination of protein (Shi *et al.*, 2013 ▸, Xu *et al.*, 2019 ▸) or organic small molecule structures (Jones *et al.*, 2018 ▸; van Genderen *et al.*, 2016 ▸).

Each of these advances has revealed novel structures: G-protein coupled receptors first determined at microfocus beamlines (Rasmussen *et al.*, 2007 ▸), cell-grown crystals interrogated by X-ray free-electron laser beams (Sawaya *et al.*, 2014 ▸), whilst MicroED has revealed high-resolution structures of the toxic cores of many amyloidogenic proteins (Rodriguez *et al.*, 2015 ▸; Sawaya *et al.*, 2016 ▸). MicroED has also proven to be a powerful method for the interrogation of small molecule structures, revealing atomic structures from seemingly amorphous powders (Jones *et al.*, 2018 ▸). Meanwhile, electron nanobeams (Zuo & Spence, 2017 ▸) ∼2–150 nm in size can facilitate diffraction from challenging beam-sensitive materials such as zeolites (Smeets *et al.*, 2018 ▸), polymers (Panova *et al.*, 2016 ▸, 2019 ▸), organic small molecules (Brázda *et al.*, 2019 ▸) and proteins (Lanza *et al.*, 2019 ▸; Bücker *et al.*, 2020 ▸), as well as more radiation-hardy inorganic materials (Mugnaioli *et al.*, 2018 ▸).

Capitalizing on innovations in electron nanodiffraction (Eggeman *et al.*, 2015 ▸; Meng & Zuo, 2016 ▸), we demonstrate the collection of high-resolution tomographic diffraction tilt series from single crystals using electron nanobeams with a full width at half-maximum of ∼12 nm. Scanning nanobeam electron-diffraction tomography (NanoEDT) data are collected by coupling four-dimensional scanning transmission electron microscopy (4D-STEM) strategies (Ophus, 2019 ▸; Gallagher-Jones *et al.*, 2019 ▸) with sample tilting along one or more axes. Meaningful diffraction signals are measured using a hybrid-counting strategy implemented on sparse data collected using direct electron detectors. Data are reduced from digitally selected areas of a scan to determine the structure of a six-residue segment from the OsPYL/RCAR5 protein, a positive regulator of abscisic acid signal transduction in seed germination and early seedling growth from *Oryza sativa*. The determination of this peptide structure by NanoEDT severs our need for a pre-defined diffraction aperture during data collection and opens a new realm of possibilities for structure determination from arbitrarily defined nanocrystalline regions.

## 2. Methods   

### 2.1. Crystallization of AVAAGA   

Crystals of AVAAGA peptide, whose sequence was derived from residues 24–29 of OsPYL/RCAR5 (LOC_Os5g12260.1), were grown using the hanging-drop method. Lyophilized OsPYL/RCAR5 peptide [>98% purity by high-pressure (high-performance) liquid chromatography, GenScript] was dissolved in double distilled deionized water to a final concentration of 10 mg ml^−1^. Two microlitres of the peptide were mixed with 2 µl of a well solution, consisting of 10% EtOH, on a glass slide and equilibrated against 500 µl of well solution over a 24-well plate. High-quality needle-shaped nanocrystals formed in 1–2 d.

### 2.2. Sample preparation for diffraction experiments   

Three microlitres of a crystal suspension were dispensed onto 400-mesh lacey carbon grids (Ted Pella) coated with either graphene oxide or 2 nm carbon films (UC type A) and allowed to adsorb for two minutes before blotting excess solution and allowing to air dry.

### 2.3. Collection of MicroED and discrete-angle selected-area diffraction data   

Electron diffraction was carried out on a Tecnai F30 microscope operating at 300 kV. MicroED data were collected at liquid nitro­gen temperatures whilst discrete-angle tilt-series diffraction was collected at room temperature. For MicroED data collection, a suitable crystal was identified in over-focused diffraction mode. The crystal was then isolated using a 1 µm selected-area aperture and continuously rotated between 45 and −45° at a rate of 0.3° s^−1^ whilst being continuously illuminated by the electron beam. Diffraction frames were recorded as a movie using a TemCam-XF416 camera (TVIPS) with each frame corresponding to a 3 s exposure. For discrete-angle title-series diffraction data collection, crystals were identified and isolated in a similar manner. Crystals were rotated between 45 and −45° in discrete 1° steps, and a 3 s exposure was recorded by the camera at each angular step. All measurements were performed at spot size 11 with the C2 lens set at 57% to ensure a low dose of ∼0.01 e^−^ Å^−2^ s^−1^ or ∼3 e^−^ Å^−2^ per dataset.

### 2.4. Collection of NanoEDT data   

Data collection for NanoEDT was performed on the TEAM I microscope at Lawrence Berkeley National Laboratory operating at an accelerating voltage of 300 kV in microprobe STEM mode. The probe was focused to a size of ∼12 nm with a semi-convergence angle of 0.09 mrad utilizing a 5 µm C2 aperture (see Fig. S1 in the Supporting information). Samples were first located using a coarse STEM scan. Once a suitable crystal was located, the focused probe was raster scanned across the crystal with a step size of 40 nm covering a total area of ∼1 by 3 µm. Data were recorded on a Gatan K2-IS direct electron detector operating at 400 frames s^−1^, with each frame representing a single scan point. After each scan was completed the sample was rotated by 1° along the holder axis and the process was repeated to give a final angular range of 45 to −45° tilt. The total dataset then consisted of 30 by 70 by 90 individual diffraction patterns. Samples were maintained at liquid nitro­gen temperature throughout data collection in a Gatan 636 holder. The total dose per frame was ∼1 e^−^ Å^−2^. The total accumulated dose is mitigated by having a step size significantly coarser than the probe size, thus reducing the likelihood that a specific sample volume will be illuminated with the same beam intensity at every tilt angle.

### 2.5. Processing and data reduction of continuous-rotation MicroED and discrete-angle tilt-series selected-area diffraction patterns   

MicroED data were converted to SMV format using the *tvips-tools* software package (Hattne *et al.*, 2015 ▸). The discrete-angle tilt-series selected-area diffraction dataset was converted from TIFF to binary files using a custom script in *MATLAB*. All data were indexed and integrated using *XDS* (Kabsch, 2010 ▸) and merged using *XSCALE*.

### 2.6. Processing and data reduction of NanoEDT data   

Raw data were first read into memory and pre-processed as previously described (Gallagher-Jones *et al.*, 2019 ▸). In brief, raw-data frames were aligned to a common centre using the centre of mass of the primary beam. The detector dark current was then subtracted from all frames using a median filter. A Gaussian model was fit to the distribution of pixel intensities after background subtraction to gain an estimate of the Gaussian noise of the detector. Using this model, a threshold was defined, above which single or multiple electron counts were considered to have occurred. The values were separated into counting ‘bins’ using this threshold and the recorded values were converted to ‘hybrid counts’. Hybrid counting is implemented as a means of alleviating some of the effects of coincidence loss. We have found that hybrid counting results in more accurate intensity estimates than binary counts in the presence of coincident electrons. The summation of these counts for all patterns within a single scan then represented the diffraction for that particular orientation. To ensure that only diffraction frames deriving from the crystal were included in this sum, a virtual dark-field image was reconstructed at each scan. To do this, a circular mask was defined and at each scan step all recorded electrons outside of this mask region (*i.e.* at high resolution) were integrated in a manner analogous to recording with an annular dark-field (ADF) detector. In this dark-field image, pixels representing crystal regions were significantly brighter than those of the carbon support and so could be segmented via thresholding and morphological opening/closing (Figs. S2 and S3). The indices of the segmented pixels were then used to define which diffraction patterns in the scan would be combined or excluded. The summed diffraction patterns were then converted to binary files using a custom script in *MATLAB*. Indexing and integration were performed via conventional profile fitting in *XDS* and merged using *XSCALE* (Kabsch, 2010 ▸).

### 2.7. Phasing and structure refinement   

The discrete-angle tilt-series diffraction data and the MicroED dose-series datasets, with the exception of the 12 e^−^ Å^−2^ dataset, were phased by direct methods in *SHELX* (Sheldrick, 2008 ▸) and refined using *PHENIX* (Adams *et al.*, 2010 ▸). Phases for the 12 e^−^ Å^−2^ dataset were generated using the model determined from the 9 e^−^ Å^−2^ dataset in *Phaser* and then refined in *PHENIX* (Adams *et al.*, 2010 ▸). For the NanoEDT data, initial phasing was performed by a fragment-based search method using the *ARCIMBOLDO* software equipped with a library of poly-glycine 4mers derived from amyloid peptides. A 268-member library of tetrameric poly-glycine steric zipper fragments derived from over 100 previously determined structures was used in the program *ARCIMBOLDO-BORGES* (Rodríguez *et al.*, 2009 ▸; Usón *et al.*, 2013 ▸). Fragments were individually analysed by *Phaser* rotation and translation analysis, and top-scoring chains were selected as inputs for *SHELXE* expansion by density modification and mainchain autotracing. The program was able to identify low mean phase error fragment solutions based on log likelihood gain (LLG) and initial correlation coefficient (CC), which were sufficient to provide initial phases despite failing to expand in *SHELXE* (Sheldrick, 2008 ▸). This fragment was then used as a starting point for building and refinement in *Coot* (Emsley *et al.*, 2010 ▸) and *PHENIX* (Adams *et al.*, 2010 ▸), respectively. Whilst the majority of datasets were determined to be space group 19 (*P*2_1_2_1_2_1_), in some of the MicroED datasets we noted a potential ambiguity in the space-group assignments to one of lower symmetry (space group 4, *P*2_1_), with all unit-cell dimensions the same except for β which was 91 instead of 90°. To address this ambiguity, we first re-indexed the data in space group 1 and ran the program *POINTLESS* to assess the Laue group symmetry and identify systematic absences. The Laue group was determined to be *mmm* with a probability of 97%, suggesting that the space group should be orthorhombic. Systematic absences were determined along the *h* = 0 axis; *k* = 0 and *l* = 0 were unfortunately in the missing wedge. Additionally, we also merged all MicroED datasets in *P*2_1_, solved the structure by direct methods and refined to a final *R*_work_/*R*_free_ of 20/23, comparable with the *P*2_1_2_1_2_1_ >space group. This solution had two chains in the asymmetric unit with an all-atom RMSD of 0.026 Å between chains, suggesting they were actually related by a symmetry operation. As a final check, we assessed the expected error in space-group assignment in *XDS* and found it to be ∼1°, large enough to explain the distortion of the β angle. Given this evidence, we chose to use the higher-symmetry space group in the rest of the analysis.

### 2.8. Estimation of crystal thickness from 4D-STEM data   

Crystal thickness was estimated using the log-ratio formula as employed in electron energy-loss spectroscopy experiments,
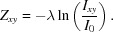
Here *Z_xy_* represents the thickness at a given pixel and λ is the mean free path of electrons through the peptide crystal, set at 332 nm as in previous experiments (Gallagher-Jones *et al.*, 2019 ▸). *I_xy_* is the transmitted intensity at a given scan position based on a combination of the integrated intensity of the central beam and the integrated intensity at Bragg peak locations identified from the aggregate diffraction pattern of the 4D-STEM scan, *i.e.* *I*_inelastic_ = *I*_0_ − (*I*_transmitted_ + *I*_elastic_). We found that omitting the electrons scattered elastically led to erroneous over-estimation of transmission loss due to inelastic scattering. *I*_0_ is estimated by taking the average value of transmitted intensity over vacuum (*i.e.* a hole in the lacey carbon) minus two times the standard deviation of the values in this region to account for fluctuations in the intensity of the electron beam. Because of the high intensity at the central beam, coincidence loss was too high to provide an accurate estimate of transmitted intensity from hybrid counts. Instead, the raw detector counts after background correction were used. The degree of coincidence in this region of the pattern depends on experiment geometry and dose. The geometry here narrowed the size of the recorded central-beam disc to the degree that coincidence was significant. Under different conditions, the intensities recorded at the central beam do not reach the saturation level of the detector (Gallagher-Jones *et al.*, 2019 ▸).

### 2.9. Tomographic reconstruction of crystals from virtual dark-field images   

Reconstructed maps of crystal thickness, as described above, were first roughly aligned to a common tilt axis using features of the lacey carbon substrate. Because of sample drift during data collection, the images were cropped to remove any regions of the crystal that were not consistently in the field of view throughout the entire tilt series. A flat background was calculated from regions of the images that sampled vacuum and subtracted, and any resulting negative values were set to zero. Tomographic reconstruction was performed using the *GENFIRE* algorithm with image-shift refinement (Pryor *et al.*, 2017 ▸). Reconstructed volumes were visualized with *Chimera* (Pettersen *et al.*, 2004 ▸).

### 2.10. Comparison of integrated intensities   

Intensities recorded by either (1) NanoEDT and MicroED, (2) NanoEDT and discrete-angle diffraction or (3) MicroED and discrete-angle diffraction were merged together using *SCALEPACK* (Minor *et al.*, 2006 ▸) to ensure that only reflections measured by both methods were compared in subsequent analysis. Fourier magnitude plots were created of all reflections with an *I*/σ above 2, and linear regression was performed in *MATLAB*. Comparisons of zone-axis reflections were performed using *VIEWHKL* (Winn *et al.*, 2011 ▸).

### 2.11. Peak identification in multi-pattern data   

To enhance the contrast of the multi-pattern diffraction patterns the data were binned by a factor of five. Peaks were then localized via template matching with a circular template six pixels in diameter using normalized cross correlation implemented in *MATLAB*.

## 3. Results   

### 3.1. Collecting nanobeam electron-diffraction tomograms from OsPYL/RCAR5 peptide nanocrystals   

**Figure 1:**
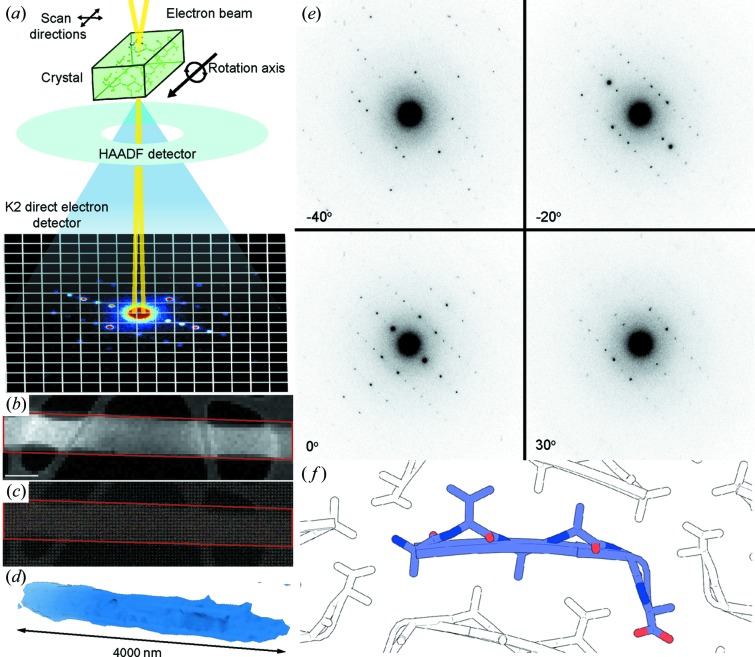
Overview of the scanning NanoEDT experiment. (*a*) Schematic of the experimental geometry for collecting nanobeam electron-diffraction data with key components highlighted. (*b*) ADF image of a crystal of segment ^24^AVAAGA^29^ from the OsPYL/RCAR5 protein interrogated by an electron beam. The scale bar represents 400 nm. (*c*) Composite image of all the diffraction patterns collected simultaneously with the ADF image in (*b*). The red outline indicates the region of the image used to compute diffraction patterns. (*d*) Tomographic reconstruction of the crystal in (*b*). (*e*) Examples of diffraction images taken at discrete orientations during electron-diffraction tomography. (*f*) Atomic structure of the OsPYL/RCAR5 peptide ^24^AVAAGA^29^ solved by NanoEDT.

To assess whether meaningful diffraction could be collected from nanometre-size regions of a crystal by NanoEDT, we scanned a focused electron beam of 12 nm in diameter (Fig. S1) through crystals of the OsPYL/RCAR5 peptide. The crystals were needle shaped (Figs. S2 and S3), ∼360 nm thick, 500 nm wide and several micrometres in length [Figs. 1 ▸(*d*) and S4]. In NanoEDT, tilt series were collected as consecutive scans at specified angles, typically separated by 1 to 2° increments (Figs. S2 and S3). Each scan grid had a spacing of 40 nm covering a total area of 1 by 4 µm [Figs. 1 ▸(*b*) and 1(*c*)]. The spacing in our scans ensured minimal probe overlap between adjacent illuminated areas in each scan, thus limiting the total dose imparted across the crystal.

Because of the small number of unit cells illuminated, individual diffraction images collected on the K2-IS at 400 frames s^−1^ were sparse. To extract the most meaningful signal from these data we employed an *ex post facto* electron-counting algorithm that converted the integrated detector signal to hybrid counts (Gallagher-Jones *et al.*, 2019 ▸). Hybrid counting offers the benefits of increased sensitivity to weak signals achieved by electron counting, whilst maintaining some of the dynamic range lost because of coincident electrons. The diffraction patterns from a single scan at a single crystal orientation were then computationally combined to produce a single diffraction pattern that represented the sum of all electron counts across a defined region of the scan [Fig. 1 ▸(*e*)].

Exploiting NanoEDT’s ability to construct a real-space image from the diffraction data, we digitally selected diffraction from a specified region of a crystal or field of view within a scan. Regions of interest were identified from the ADF image acquired simultaneously with the diffraction patterns or from a reconstructed virtual dark-field image. Diffraction signal was then selected only from these regions to produce a set of diffraction patterns representing a tilt series. Collectively, the chosen regions outlined the distinguishable bounds of the crystal [Figs. 1 ▸(*b*), 1 ▸(*c*), S2 and S3]; their dimensions matched those obtained from three-dimensional tomographic reconstructions of target crystals based on estimates of their thickness (Figs. S4 and S5). By rotating the sample stage 1° between scans, we computed 81 summed diffraction patterns spanning an angular range of ±40° (Fig. 1 ▸). The nominal exposure over the full rotation series was ∼81 e^−^Å^−2^, within the range of a typical cryo tomography experiment (Mahamid *et al.*, 2016 ▸).

### 3.2. Structure of an OsPYL/RCAR5 peptide determined by NanoEDT   

**Figure 2:**
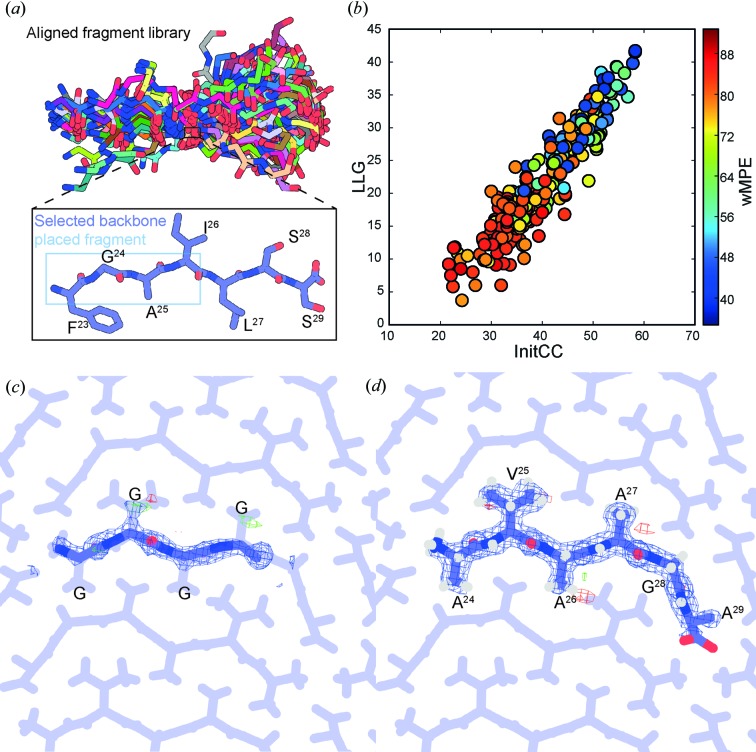
Fragment-based phasing of NanoEDT data. (*a*) The amyloid peptide fragment library used as input for *ARCIMBOLDO*. The final fragment placed and the structure it is derived from (Sawaya *et al.*, 2016 ▸) are highlighted by the blue and black boxes, respectively. (*b*) LLG versus initial CC for all fragments used by *ARCIMBOLDO* to find the initial phasing solution. The colour bar represents the mean phase error of a given fragment compared with the final solution. (*c*) The initial fragment placed by *ARCIMBOLDO* (blue) overlaid on the final solution (purple). (*d*) The final refined structure of the OsPYL/RCAR5 peptide ^24^AVAAGA^29^. H atoms are shown in white and highlighted by black arrows. The blue mesh represents the 2*F*_o_ − *F*_c_ map (contoured at 1σ) and the green/red mesh represents the *F*_o_ − *F*_c_ map (contoured at ±3σ).

We indexed and integrated NanoEDT data from regions of interest in two different crystals of the OsPYL/RCAR5 peptide and then assembled tilt series from each crystal into a three-dimensional reciprocal lattice. The outermost reflections observable in each tilt series correspond to ∼1.1 Å resolution (Fig. S6) and the two datasets were merged to give a final set of reflections with an overall completeness of 70% at 1.35 Å (Table 1 ▸). While the diffracted signal at high resolution was not sufficiently complete for direct methods, *ab initio* fragment-based phasing using the program *ARCIMBOLDO* was successful in generating initial phases from a library of probes consisting of poly-glycine tetramers [Fig. 2 ▸(*a*)]. A single four-residue β-strand was placed by *ARCIMBOLDO* (Rodríguez *et al.*, 2009 ▸; Usón *et al.*, 2013 ▸) with an LLG of 35.9 and an initial CC of 55.49 [Figs. 2 ▸(*b*) and 2(*c*)]. This was subsequently built and refined using electron atomic scattering factors in *PHENIX* (Adams *et al.*, 2010 ▸) [Fig. 2 ▸(*d*)] to produce a class 4 amyloid zipper (Sawaya *et al.*, 2007 ▸) with six residues per strand. The overall structure had *B* factors that were sufficiently low (1–5 Å^2^) to detect H atoms for many of the residues at the core of the zipper [Fig. 2 ▸(*d*)]. Inclusion of H atoms in the refinement dropped the crystallographic *R* factors from 0.270/0.310 to their final values of 0.253/0.260; this was considered a significant-enough difference to warrant their inclusion. These *R*_work_/*R*_free_ values are consistent with structures solved by MicroED with a comparable level of electron exposure (Table 1 ▸). Residues at the C terminus showed considerably higher *B* factors than the rest of the structure resulting in less well defined density in this region [Fig. 2 ▸(*d*)]. We initially attempted to model H atoms at this position; however in doing so, the *R* factors slightly increased and the density around the C_α_ carbon significantly depreciated leading us to exclude these H atoms in the C-terminus of the final model.

### 3.3. Comparison of intensities measured by different electron-diffraction methods   

**Figure 3:**
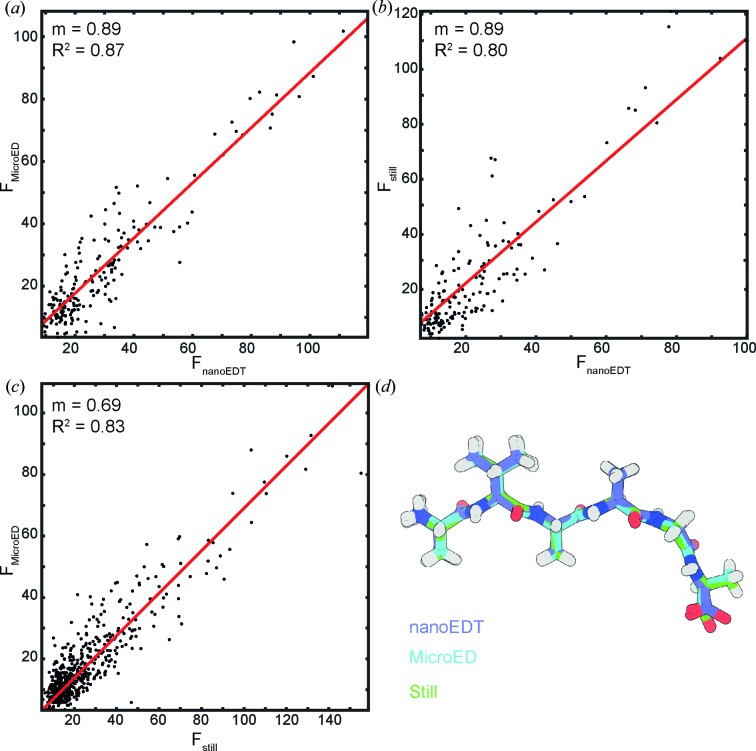
Pairwise comparison of Fourier magnitudes of OsPYL/RCAR5 peptide ^24^AVAAGA^29^ crystals recorded by different methods. (*a*) Linear-regression fit to the pairwise comparison of Fourier magnitudes collected using MicroED and NanoEDT. (*b*) Linear-regression fit to the pairwise comparison of Fourier magnitudes collected using fixed-angle diffraction and NanoEDT. (*c*) Linear-regression fit to the pairwise comparison of Fourier magnitudes collected using MicroED and fixed-angle selected-area diffraction. (*d*) Alignment of the structures determined by each of the three methods. The all-atom RMSD is <0.15 Å

We assessed the accuracy of intensities measured by NanoEDT by comparing them to intensities measured by selected-area electron-diffraction approaches: either using continuous-rotation or discrete-angle tilt-series diffraction. We determined structures of the OsPYL/RCAR5 peptide from diffraction collected by continuously rotating crystals in an electron beam (MicroED) and by capturing diffraction at fixed angles in discrete 1° increments from crystals whilst exposing them to a 300 kV electron beam. All the experiments were performed on crystals of the OsPYL/RCAR5 peptide from the same batch condition and prepared in the same way; in all cases the angular sampling was ±45°. Structures were determined by direct methods from two different datasets: merged MicroED data from three crystals and discrete-angle diffraction recorded from a single crystal. Comparison of the structures from all three datasets showed a high degree of similarity with an overall all-atom RMSD of 0.145 ± 0.03 Å, with the greatest deviation occurring at the C terminus [Fig. 3 ▸(*d*)]. The overall statistics of the refinements are summarized in Table 1 ▸. The best-quality data were obtained by merging MicroED diffraction data from several crystals, as reflected in the final refined *R* factors. Interestingly, we note that the *R* factors observed from both NanoEDT data and discrete-angle tilt-series diffraction are similar to those obtained from conventional MicroED data despite potential issues with partiality and coincidence loss (Table 1 ▸).

To assess the extent of coincidence loss in NanoEDT datasets, we analysed the distribution of hybrid counts within a 4D-STEM scan for the central beam and three different types of reflections: a high-intensity reflection, an intermediate intensity reflection and a low-intensity reflection (Fig. S7). The number of coincident electrons seems to be low within the Bragg reflections as all reflections had a similar distribution of counts, with the majority being single counts. By contrast, the count distribution for the central beam skews towards higher counts suggesting a higher degree of coincidence loss in this intense region. This is best shown by comparison of maps of integrated intensity at the central-beam position using traditional integration and after conversion to hybrid counts (Fig. S8). The maps calculated from hybrid-counting data appear much flatter and do not reflect the true pattern of transmission, suggesting issues with coincidence loss at the central beam. To further explore differences between the various electron-diffraction datasets, we performed pairwise comparisons of the magnitudes from each dataset after scaling their intensities. We performed linear regression on these comparisons to visualize and quantify the correlation between datasets [Figs. 3 ▸(*a*)–3(*c*)]. Overall, the discrete-angle tilt-series diffraction data had the poorest correlation to all other datasets, with the highest correlation being between the data taken by conventional MicroED and NanoEDT [Figs. 3 ▸(*a*)–3(*c*)]. We note, however, that since the discrete-angle data was obtained from a single crystal and at room temperature, some of this difference can be attributed to crystal-to-crystal variation and the more rapid decay of intensities caused by radiation damage at 293 K. Visual inspection of the distribution of Bragg peak intensities across the three principle zone axes in all datasets supported this high degree of similarity (Fig. S5). However, comparisons along the *h* = 0 and *k* = 0 zone axes were limited by the narrow wedge of data collected by NanoEDT, exacerbated by the orientation bias of OsPYL/RCAR5 peptide crystals on the grid in these experiments. Some slight intensity differences can be noted along the major zones for the discrete-angle and NanoEDT datasets (Fig. S9) that may be the result of dynamical scattering and/or the lack of full angular integration in these experiments. These deviations are small enough to still allow refinement of the crystal structure from the integrated intensities.

### 3.4. Impact of electron exposure on peptide structures in NanoEDT   

**Figure 4:**
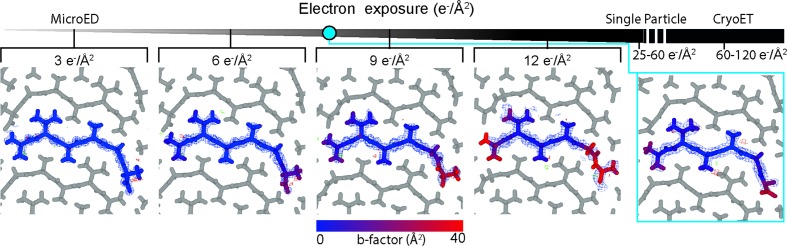
Estimation of electron exposure in NanoEDT. The top gradient represents increasing exposure to the incident electron beam. Several cryoEM methods are highlighted with typical values of exposure. The blue dot indicates the apparent exposure of the NanoEDT structure based on comparison with observed *B* factors in structures solved by MicroED at a known electron exposure. The blue mesh represents the 2*F*_o_ − *F*_c_ map (contoured at 1σ).

Although the integrated electron fluence per illuminated region in NanoEDT is considerably higher than in MicroED, the observed impacts of its higher exposure on the final structure determined by NanoEDT are more consistent with a conventional MicroED exposure (Hattne *et al.*, 2018 ▸). To evaluate the impact of electron exposure during NanoEDT data collection, we compared our NanoEDT structure of the OsPYL/RCAR5 peptide with those determined by MicroED under various exposures at cryogenic conditions to a 300 kV electron beam. To observe the effect of increasing electron exposure on OsPYL/RCAR5 peptide crystals, we collected four consecutive datasets from three different crystals with a total estimated exposure of 3 e^−^ Å^−2^ per dataset. Merging the data from three different crystals allowed us to determine MicroED structures of OsPYL/RCAR5 peptide with a collective exposure of 3, 6, 9 or 12 e^−^ Å^−2^ (Fig. 4 ▸). Merging data from several crystals was necessary to reduce the impact of completeness and detector noise on structure refinement, reducing the uncertainty in any observations of radiation damage (see Table S1 in the Supporting information).

We observed that as exposure increases, there is a proportionate increase in the *B* factors of the atoms at the C-terminus of the OsPYL/RCAR5 peptide structure. This was coupled with an overall loss of resolvable density in this region (Fig. 4 ▸). By the time the crystals had been exposed to 9 e^−^ Å^−2^, the OsPYL/RCAR5 peptide structure showed no visible density for C-terminal oxygen. Because the OsPYL/RCAR5 peptide structure determined by NanoEDT shows a *B* factor profile in between the 6 and 9 e^−^Å^−2^ exposure structures in the MicroED dose series, we believe the effective dose experienced by the crystals in the NanoEDT structure is consistent with an effective exposure of 6 to 9 e^−^Å^−2^ (Fig. 4 ▸).

### 3.5. Achieving diffraction pattern separation in multi-crystal fields of view   

**Figure 5:**
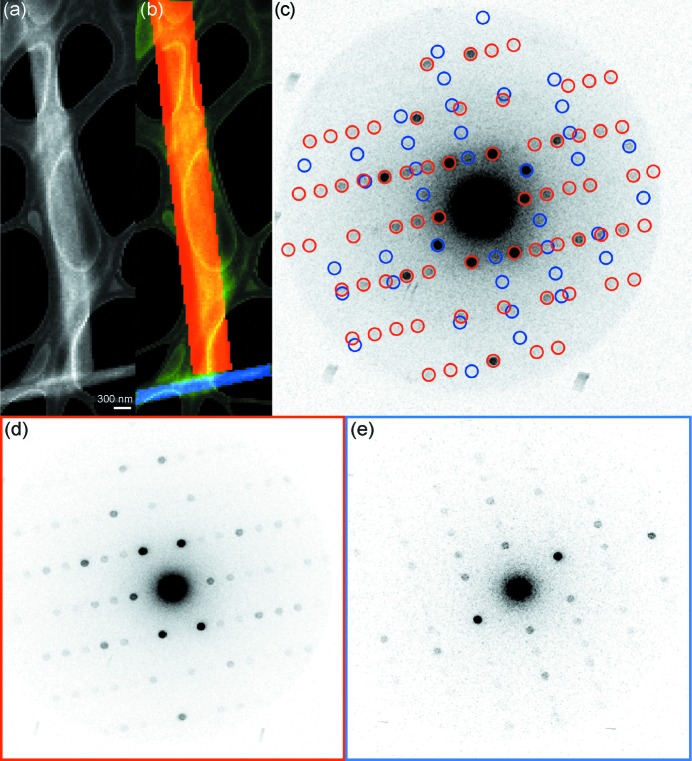
Digital separation and extraction of multiple diffraction patterns from separate crystals in a single field of view. (*a*) An ADF image of two OsPYL/RCAR5 peptide crystals. (*b*) Segmentation of the two crystals from (*a*). (*c*) A 4D-STEM pattern calculated from the entire field of view in (*a*). Bragg reflections arising from the masked regions in (*b*) are highlighted by circles of their respective colour. (*d*) A 4D-STEM pattern calculated from only diffraction patterns captured from the red region in (*b*). (*e*) A 4D-STEM pattern calculated from only diffraction patterns captured from the blue region in (*b*).

The ability to digitally define regions of interest using NanoEDT extends to polycrystalline samples and clustered crystals, from which coincident reciprocal lattices can be separated yielding high-resolution single-crystal diffraction (Fig. 5 ▸). This is achieved by integrating diffracted signal from separate regions within adjacent crystallites, allowing the identification of each reciprocal lattice within a multi-lattice diffraction pattern (Fig. 5 ▸). This approach relies on spatial separation of crystallite regions in ADF images or simulated dark-field images of a grid region, and thus avoids the need for lattice deconvolution or multi-lattice indexing (Gildea *et al.*, 2014 ▸).

## 4. Discussion   

In a first demonstration of the powerful application of NanoEDT, we determined the atomic structure of an amyloid-forming OsPYL/RCAR5-derived peptide phased by fragment-based methods. We demonstrated the capture of meaningful diffraction from regions of a peptide crystal as small as 40 nm and combined this data digitally post-experiment. Subsequent data reduction allows for structural determination and refinement from user-selected areas of single or clustered nanocrystals. Structures determined by NanoEDT are accurate, comparing favourably with structures of the same sample determined by selected-area diffraction methods, and have refinement statistics comparable with those from other methods (Table 1 ▸). However, NanoEDT allows atomic detail to be extracted from a digitally defined nanoscale volume.

The general applicability of NanoEDT to various nanocrystalline substrates is limited only by their diffracting quality at the 10–40 nm scale, which matches the beam sizes and grid samplings demonstrated in our experiments. Our current efforts correspond to observations from a single peptide but the methods implemented could benefit a broad variety of nanocrystalline samples with an equivalent or greater tolerance to electron exposure. We note that the estimated electron exposure (∼81 e^−^ Å^−2^) is far greater than the impact observed on the structure determined by NanoEDT, which corresponded best with MicroED structures irradiated tenfold less. We rationalize this by noting that since scan points were 40 nm apart on a regular grid, the crystalline area mapped in a single scan step (1600 nm^2^) is ∼14 times larger than the area directly illuminated by the electron beam (113 nm^2^). Thus, the actual accumulated exposure at the illuminated regions may be near 81 e^−^ Å^−2^, while the average exposure across the entire crystal is likely to be an order of magnitude lower. This is shown by the high-resolution diffraction detected near the end of the NanoEDT tilt series, which did not present an attenuation of diffracted signal commensurate with such a high electron exposure. In fact, in conventional MicroED experiments (Hattne *et al.*, 2018 ▸), significant radiation damage has been observed at electron exposures as low as 3–10 e^−^ Å^−2^.

We envision that integration of currently available hardware and software improvements, including expanded angular sampling, cryogenic preservation procedures, precession of the probe and automation of crystal tilting, will greatly enhance the quality of data obtained by NanoEDT. Given the already high correlation of NanoEDT data to that collected by conventional MicroED methods, we see no absolute hinderance to the selective inclusion of diffraction from digitally defined regions of a sample. The similarity between NanoEDT and continuous-rotation electron-diffraction data (Fig. 4 ▸) indicates that NanoEDT may benefit from lattice variation due to nanocrystal bending. In previous experiments, orientation changes on the order of 1–3° have been detected over distances of 1–2 µm within single nanocrystals (Gallagher-Jones *et al.*, 2019 ▸). Averaging nanodiffraction, from different locations of a single crystal within this range, therefore represents a pseudo-rocking curve, more similar to a precession photograph than true diffraction stills.

More broadly, scanning nanodiffraction may provide a means to address some unresolved questions about sources of error in electron diffraction. There currently exists a gap between theoretical simulations of electron-diffraction phenomena and experimental observations, from the upper limit of crystal thickness for electron diffraction (Subramanian *et al.*, 2015 ▸) to the better understanding and application of dynamical scattering (Gemmi & Lanza, 2019 ▸). In practice, intensities accurate enough to solve structures by direct methods have been collected from crystals of 200–500 nm thickness (Sawaya *et al.*, 2016 ▸). Several ideas have been proposed to account for this discrepancy including: sample bending (Subramanian *et al.*, 2015 ▸; Gallagher-Jones *et al.*, 2019 ▸), mosaicity (Nederlof *et al.*, 2013 ▸), and contributions from inelastic scattering and solvent scattering (Latychevskaia & Abrahams, 2019 ▸). The true magnitude of dynamical scattering present in electron diffraction collected from crystals of macromolecules remains unclear because of the contributions of the aforementioned confounding variables. We have previously shown that through scanning nanodiffraction experiments it is possible to capture both the scale of crystal bending and variations in crystallinity and thickness within a single crystal (Gallagher-Jones *et al.*, 2019 ▸). Combining such analysis with the data-collection strategy outlined here may ultimately help decouple some of the different phenomena that occur during diffraction experiments and provide a clearer picture of the main sources of error in intensities measured by electron diffraction.

## 5. Conclusions   

Enabled by the control of nano-focused electron beams and sensitive direct electron detectors, NanoEDT has revealed the atomic structure of an amyloid-forming segment of the OsPYL/RCAR5 protein from digitally defined regions of single nanocrystals. The ability to selectively capture diffraction from digitally defined regions of a single nanocrystal or collection of nanocrystals (Fig. 5 ▸) could facilitate the unprecedented determination of atomic structures from heterogeneous or polycrystalline nanoassemblies.

## 6. Code availability   

The *MATLAB* scripts for data pre-processing of 4D-STEM data can be found at https://github.com/marcusgj13/4DSTEM_dataAnalysis. The *MATLAB* implementation of *GENFIRE* used for tomographic reconstructions can be found at https://github.com/genfire-em/GENFIRE-MATLAB.

## Supplementary Material

Supporting information. DOI: 10.1107/S2052252520004030/fq5010sup1.pdf

PDB reference: NanoEDT structure of OsPYL/RCAR5 (24–29), 6uop

PDB reference: Structure of OsPYL/RCAR5 (24–29) using discrete-angle tilt series, 6uoq

PDB reference: MicroED structure of OsPYL/RCAR5 (24–29) at 3 e^−^ Å^2^, 6uor

PDB reference: MicroED structure of OsPYL/RCAR5 (24–29) at 6 e^−^ Å^2^, 6uos

PDB reference: MicroED structure of OsPYL/RCAR5 (24–29) at 9 e^−^ Å^2^, 6uou

PDB reference: MicroED structure of OsPYL/RCAR5 (24–29) at 12 e^−^ Å^2^, 6uow

## Figures and Tables

**Table 1:** Data-collection and refinement statistics

Crystal (PDB ID)	AVAAGA (6uop)	AVAAGA (6uoq)	AVAAGA at 3 e^−^ Å^−2^ (6uor)	AVAAGA at 6 e^−^ Å^−2^ (6uos)	AVAAGA at 9 e^−^ Å^−2^ (6uou)	AVAAGA at 12 e^−^ Å^−2^ (6uow)
Data collection						
Technique	NanoEDT	Diffraction stills	MicroED	MicroED	MicroED	MicroED
Microscope	TEAM I	Technai F30	Technai F30	Technai F30	Technai F30	Technai F30
Temperature (K)	100	293	100	100	100	100
Space group	*P*2_1_2_1_2_1_	*P*2_1_2_1_2_1_	*P*2_1_2_1_2_1_	*P*2_1_2_1_2_1_	*P*2_1_2_1_2_1_	*P*2_1_2_1_2_1_
Unit-cell dimensions						
*a*, *b*, *c* (Å)	4.71, 11.49, 38.90	4.72, 11.56, 39.19	4.73, 11.32, 38.93	4.72, 11.28, 39.39	4.73, 11.36, 39.59	4.73, 11.42, 39.59
α, β, γ (°)	90.0, 90.0, 90.0	90.0, 90.0, 90.0	90.0, 90.0, 90.0	90.0, 90.0, 90.0	90.0, 90.0, 90.0	90.0, 90.0, 90.0
Resolution limit (Å)	1.35 (1.4–1.35)	1.0 (1.05–1.01)	0.9 (0.93–0.90)	0.9 (0.93–0.90)	1.0 (1.04–1.00)	1.2 (1.24–1.20)
Wavelength (Å)	0.0197	0.0197	0.0197	0.0197	0.0197	0.0197
No. of crystals merged	2	1	3	3	3	3
*R*_merge_	0.193 (0.370)	0.217 (0.357)	0.186 (0.405)	0.202 (0.666)	0.253 (0.691)	0.252 (0.696)
*R*_meas_	0.215 (0.426)	0.266 (0.440)	0.198 (0.430)	0.216 (0.706)	0.270 (0.733)	0.269 (0.739)
〈(*I*)/σ(*I*)〉	3.8 (2.1)	2.87 (1.80	6.76 (4.01)	5.57 (2.51)	4.12 (2.13)	3.74 (2.34)
CC_1/2_	0.98 (0.95)	0.96 (0.94)	0.99 (0.91)	0.98 (0.76)	0.97 (0.81)	0.98 (0.81)
Completeness (%)	68.6 (71.9)	74.4 (70.9)	97.7 (99.4)	97.8 (99.4)	97.4 (98.1)	90.0 (90.4)
No. of reflections	1981 (610)	2878 (1074)	15449 (4347)	16619 (4760)	12269 (3027)	6454 (3182)
No. of unique reflections	405 (151)	1029 (400)	1776 (468)	1792 (478)	1339 (312)	737 (339)
Multiplicity	4.9 (4.0)	2.8 (2.7)	8.7 (9.3)	9.3 (10.0)	9.2 (9.7)	8.7 (9.4)
						
Refinement						
Resolution range (Å)	5.75–1.35 (1.40–1.135)	7.5–1.0 (1.04–1.01)	7.4–0.9 (0.93–0.90)	7.4–0.90 (0.93–0.9)	7.4–1.00 (1.04–1.00)	7.5–1.20 (1.24–1.20)
No. of reflections (work)	405 (40)	1023 (85)	1768 (187)	1780 (195)	1333 (130)	731 (70)
*R*_work_	0.253 (0.397)	0.234 (0.306)	0.206 (0.302)	0.230 (0.361)	0.249 (0.367)	0.269 (0.307)
*R*_free_	0.260 (0.283)	0.256 (0.428)	0.240 (0.295)	0.244 (0.334)	0.250 (0.429)	0.358 (0.418)
CC(work)	0.948 (0.760)	0.956 (0.906)	0.953 (0.864)	0.965 (0.844)	0.962 (0.767)	0.952 (0.500)
CC(free)	0.967 (1.000)	0.969 (0.389)	0.952 (0.903)	0.966 (0.936)	0.960 (0.827)	0.904 (0.423)
No. of H atoms	30	34	34	34	34	34
No. of non-H atoms	32	32	32	32	30	30
Peptide	62	66	66	66	64	64
Water	0	0	0	0	0	0
*B* factors (Å^2^)						
Peptide	10.07	8.6	2.2	6.99	10.8	16.0
Water	N/A	N/A	N/A	N/A	N/A	N/A
RMS deviations						
RMS (bonds, Å)	0.008	0.013	0.019	0.02	0.013	0.013
RMS (angles, °)	1.064	0.891	1.218	1.2	0.79	1.01
