# Supplementary material for: Atomic structures determined from digitally defined nanocrystalline regions
Source: IUCrJ. 2020 Apr 10;7(Pt 3):490–9. doi: 10.1107/S2052252520004030 (PMC7201287; doi:10.1107/S2052252520004030)
Supplement: Supplementary file 1 [file m-07-00490-sup1.pdf]

IUCrJ

Volume 7 (2020)

Supporting information for article:

Atomic structures determined from digitally defined nanocrystalline regions

Marcus Gallagher-Jones, Karen C. Bustillo, Colin Ophus, Logan S. Richards, Jim Ciston, Sangho Lee, Andrew M. Minor and Jose A. Rodriguez

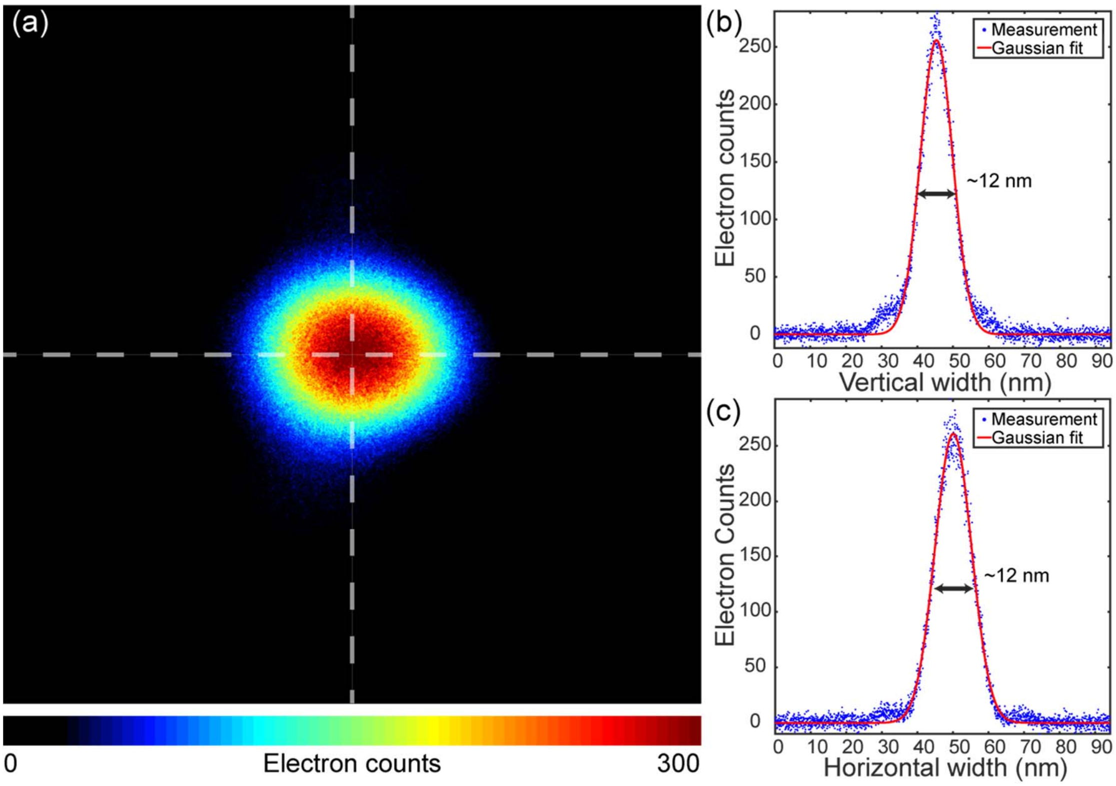

Figure S1 Analysis of the electron probe used in nanoEDT experiments. (*a*) Image of the focused probe captured on a Gatan Ultra scan 1000 with an exposure time of 3s. (*b*) Vertical cross-section of the probe (vertical white line in *a*) and Gaussian fit to the profile giving an estimated diameter of ~12 nm at FWHM. (*c*) Horizontal cross-section of the probe (vertical white line in *a*) and Gaussian fit to the profile giving an estimated diameter of ~12 nm at FWHM.

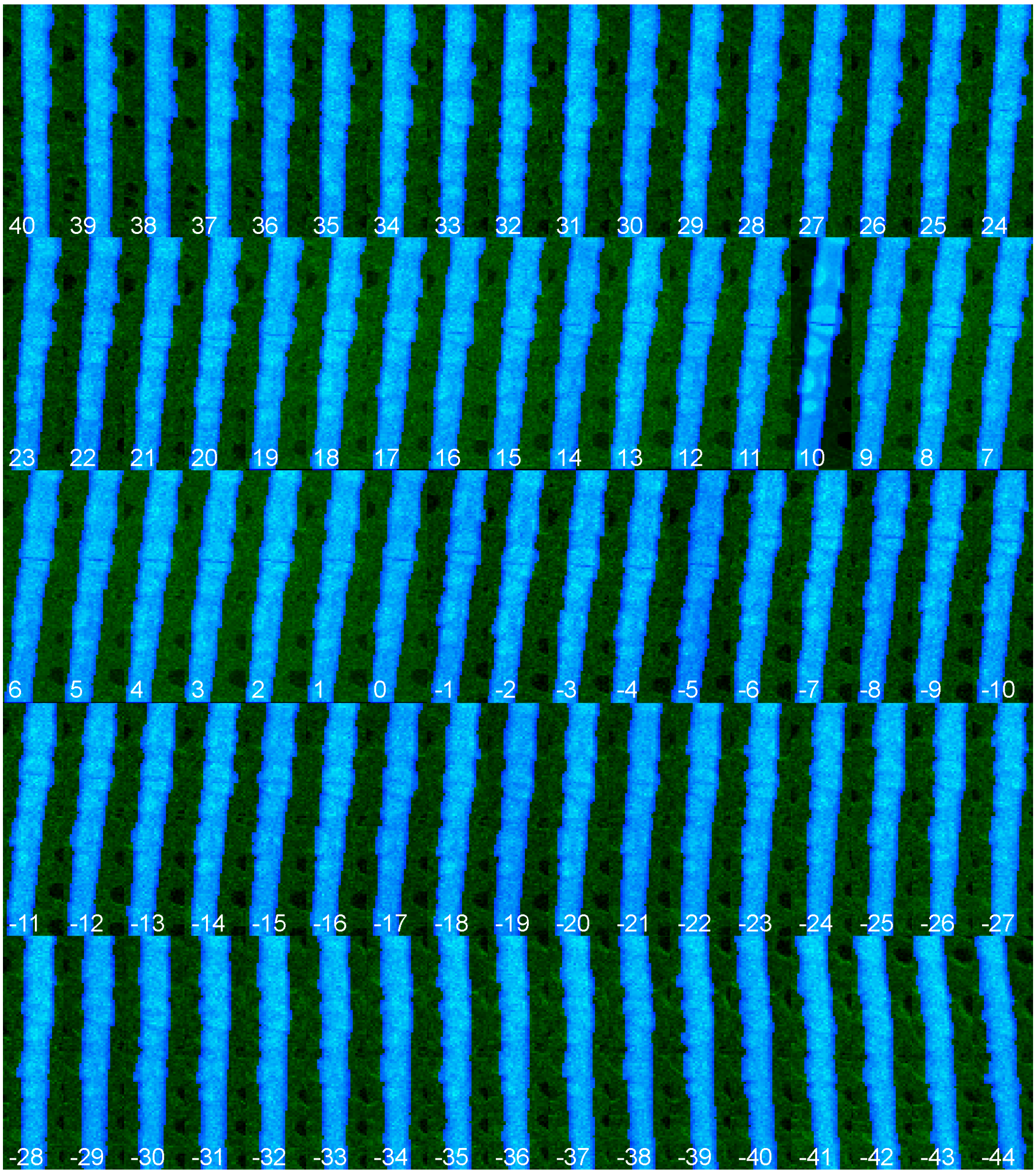

Figure S2 Digitally defined regions of nanoEDT scans taken from the first crystal. Blue pixels represent scan locations that were used to assemble diffraction patterns for the first dataset. Green and black pixels were excluded.

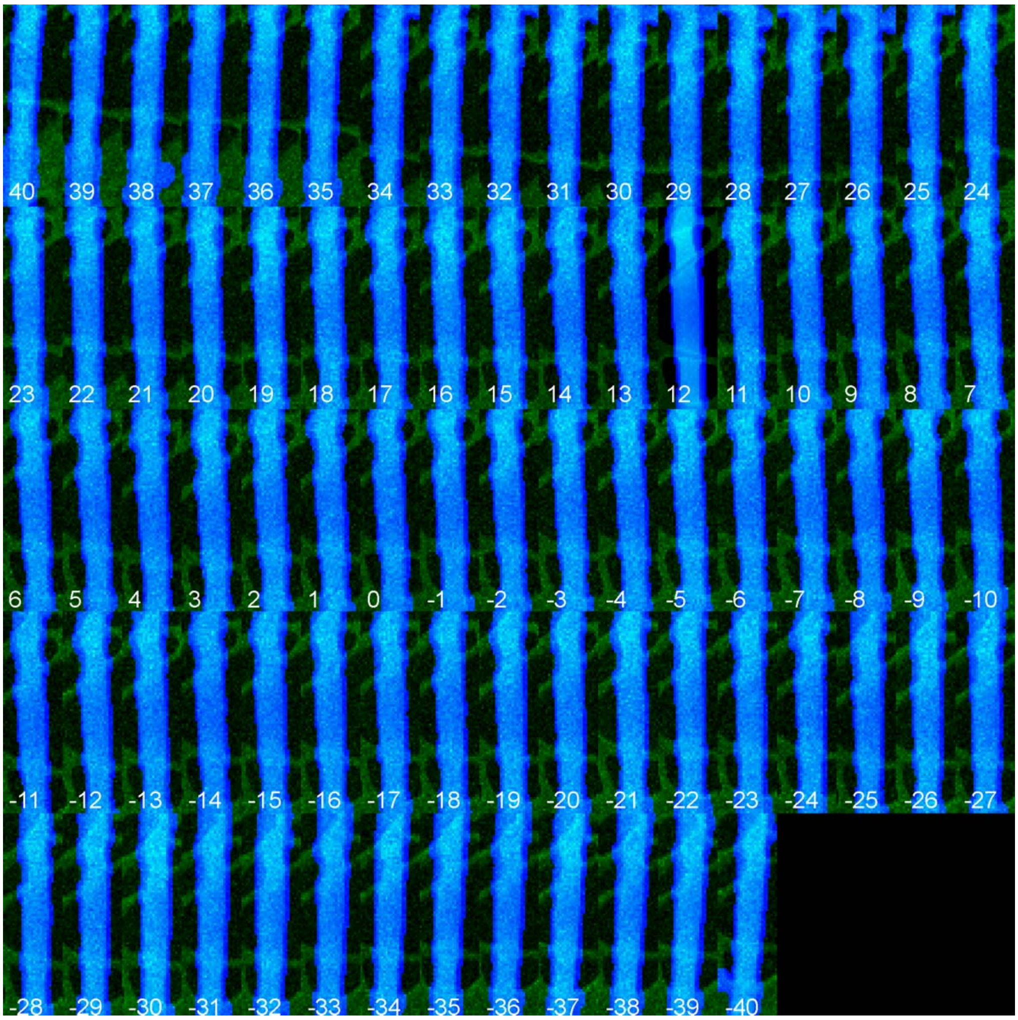

Figure S3 Digitally defined regions of nanoEDT scans taken from the second crystal. Blue pixels represent scan locations that were used to assemble diffraction patterns for the second dataset. Green and black pixels were excluded.

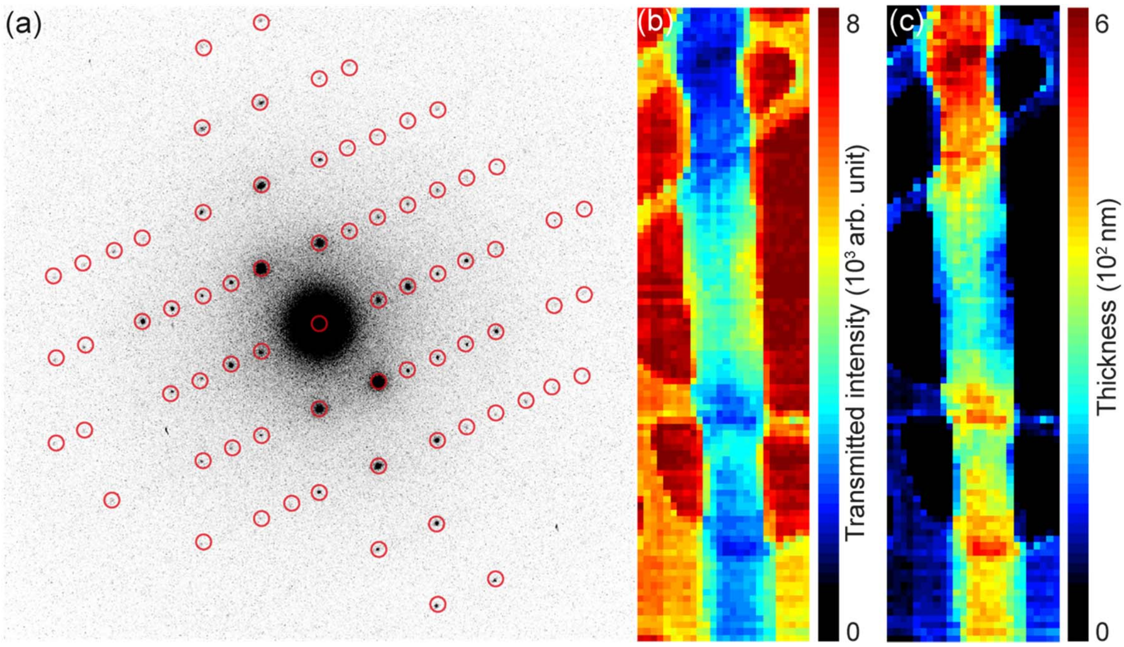

Figure S4 Estimation of crystal thickness from 4DSTEM data. (*a*) Aggregate diffraction pattern calculated from a 4DSTEM scan. Highlighted by red circles are the regions of the diffraction pattern used to estimate changes in transmission of the central beam due to inelastic scattering (I_inelastic = I_0 – (I_transmitted + I_elastic)). (*b*) Map of transmission loss due to inelastic scattering across the 4DSTEM scan calculated from highlighted regions in (*a*). (*c*) Estimated thickness of the crystal and surrounding regions based on changes in transmission in (*b*).

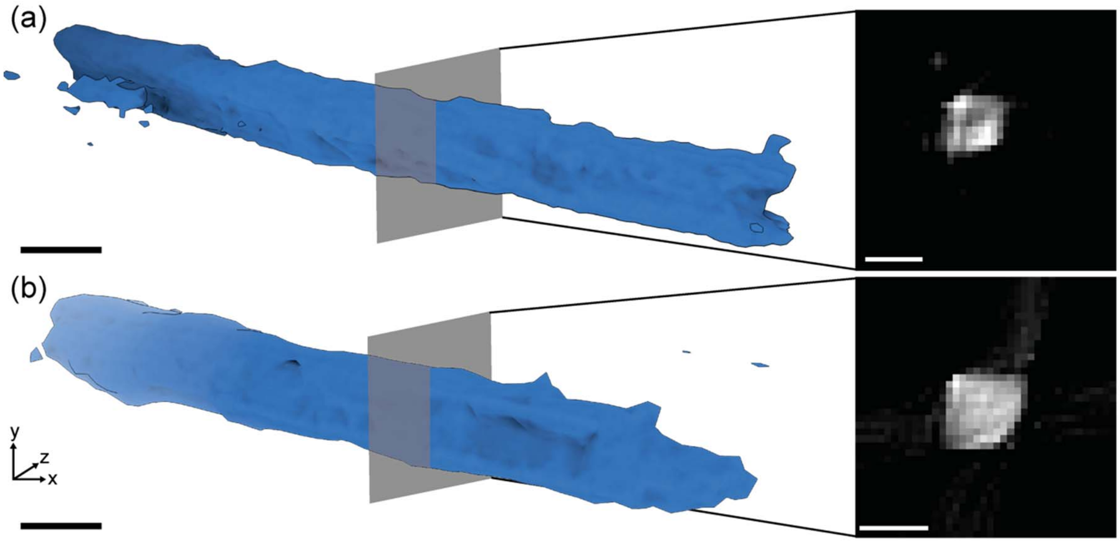

Figure S5 Tomographic reconstruction of crystals from calculated thickness maps. (*a*) Isosurface rendering of the tomographic reconstruction of the first crystal analyzed by nanoEDT in this study. The gray plane represents a slice through the reconstructed density. (*b*) Isosurface rendering of the tomographic reconstruction of the second crystal analyzed by nanoEDT in this study. The gray plane represents a slice through the reconstructed density. All scale bars represent 400 nm.

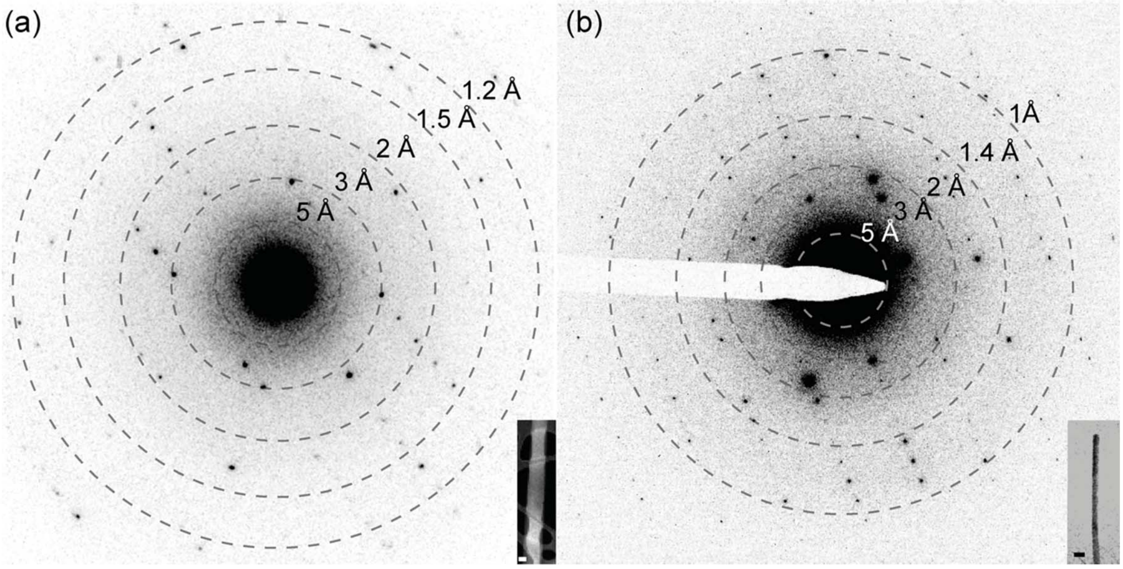

Figure S6 Diffraction patterns obtained by nanoEDT *vs.* conventional MicroED. (*a*) Computed nanoEDT diffraction pattern from 4DSTEM scans recorded on a K2-IS detector. (*b*) MicroED diffraction pattern recorded on a TemCam-XF416 CMOS detector. Inset are the crystals that the diffraction was captured from. Scalebars are 200 nm.

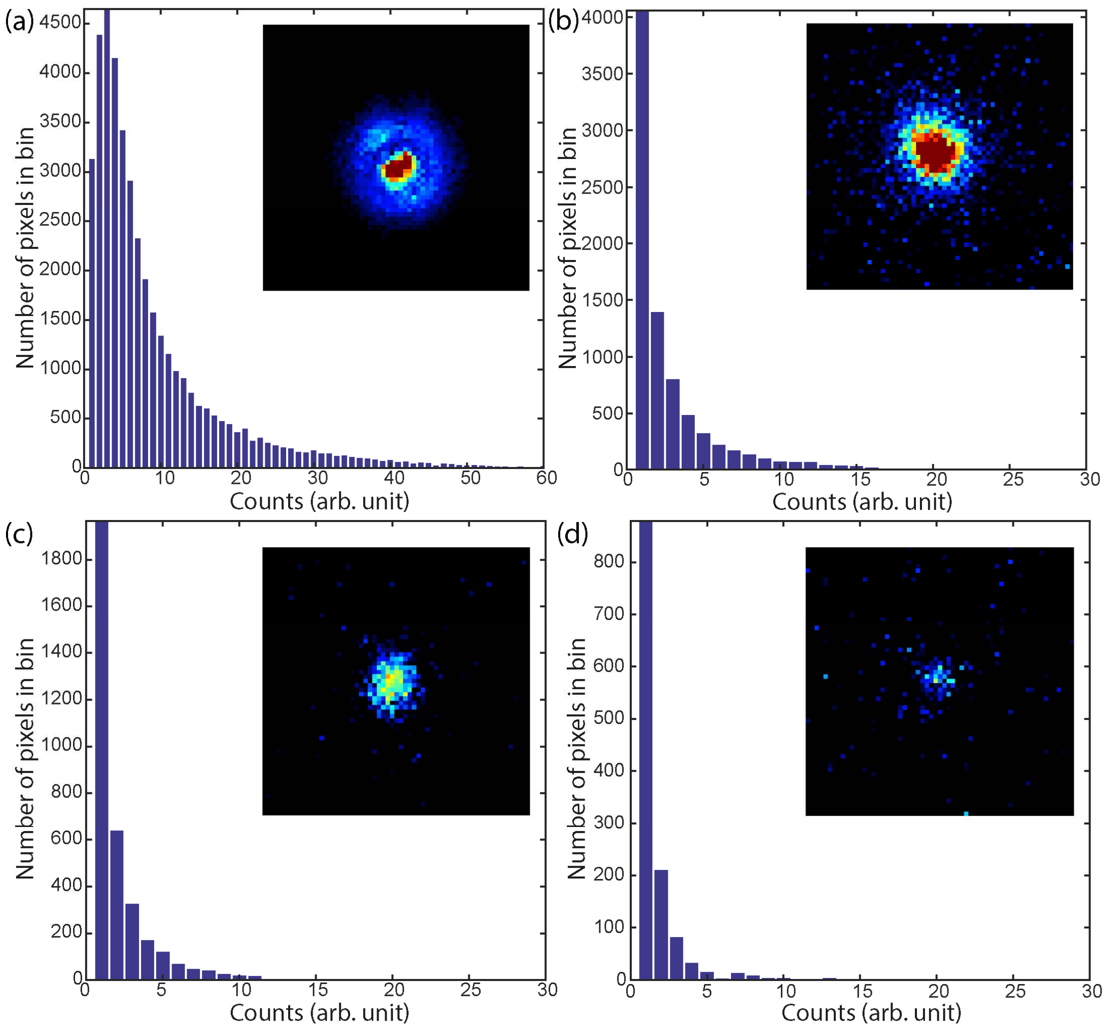

Figure S7 Comparison of hybrid count distributions in different Bragg reflections measured by nanoEDT. (*a*) Histogram of hybrid counts measured at the 000-reflection position during a 4DSTEM scan. (*b*) Histogram of hybrid counts measured at a strong reflection during a 4DSTEM scan. (*c*) Histogram of hybrid counts measured at an intermediate intensity reflection during a 4DSTEM scan. (d) Histogram of hybrid counts measured at a weak reflection during a 4DSTEM scan. Insets show the intensity profiles of the reflection in the assembled diffraction pattern. Insets in (b - d) are on the same intensity scale.

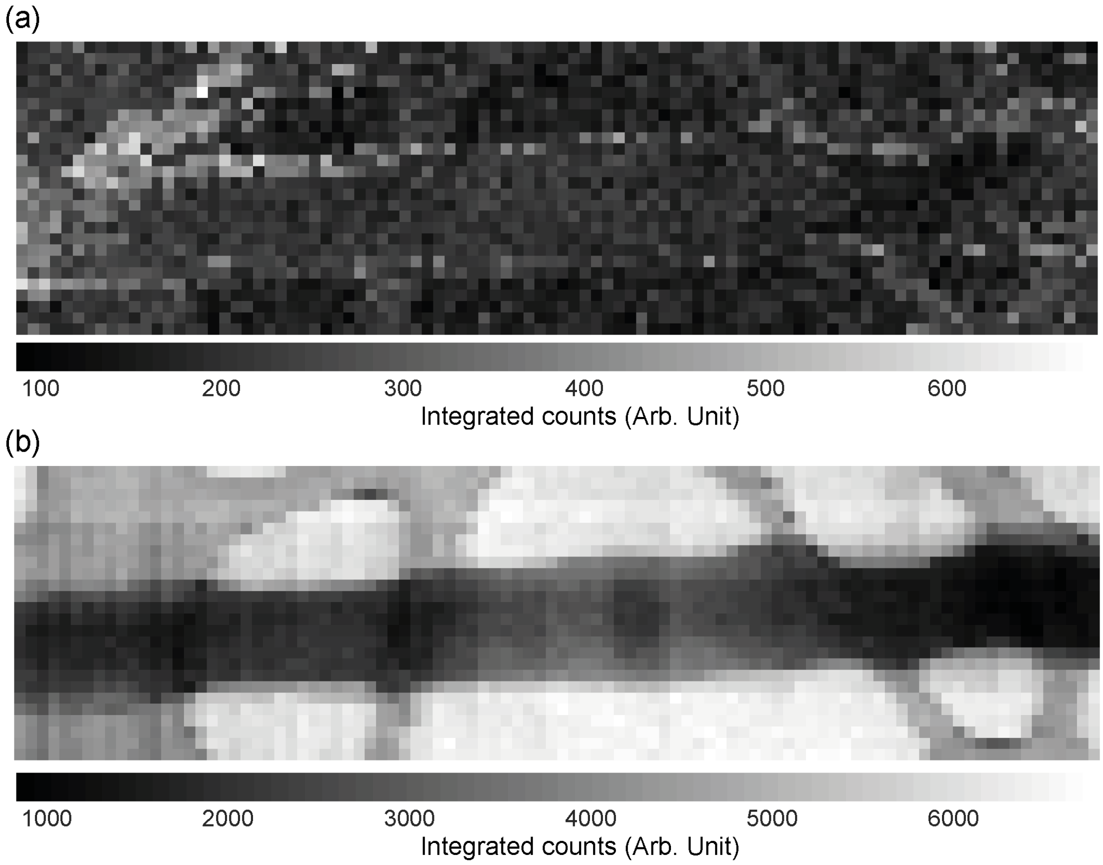

Figure S8 Comparison of transmitted intensity at the 000-peak position in 'hybrid counting' and traditional integration. (a) Map of transmitted intensity for a single 4DSTEM scan calculated after conversion to hybrid counts. (b) Map of transmitted intensity for a single 4DSTEM scan calculated from traditional integration without converting to hybrid counts.

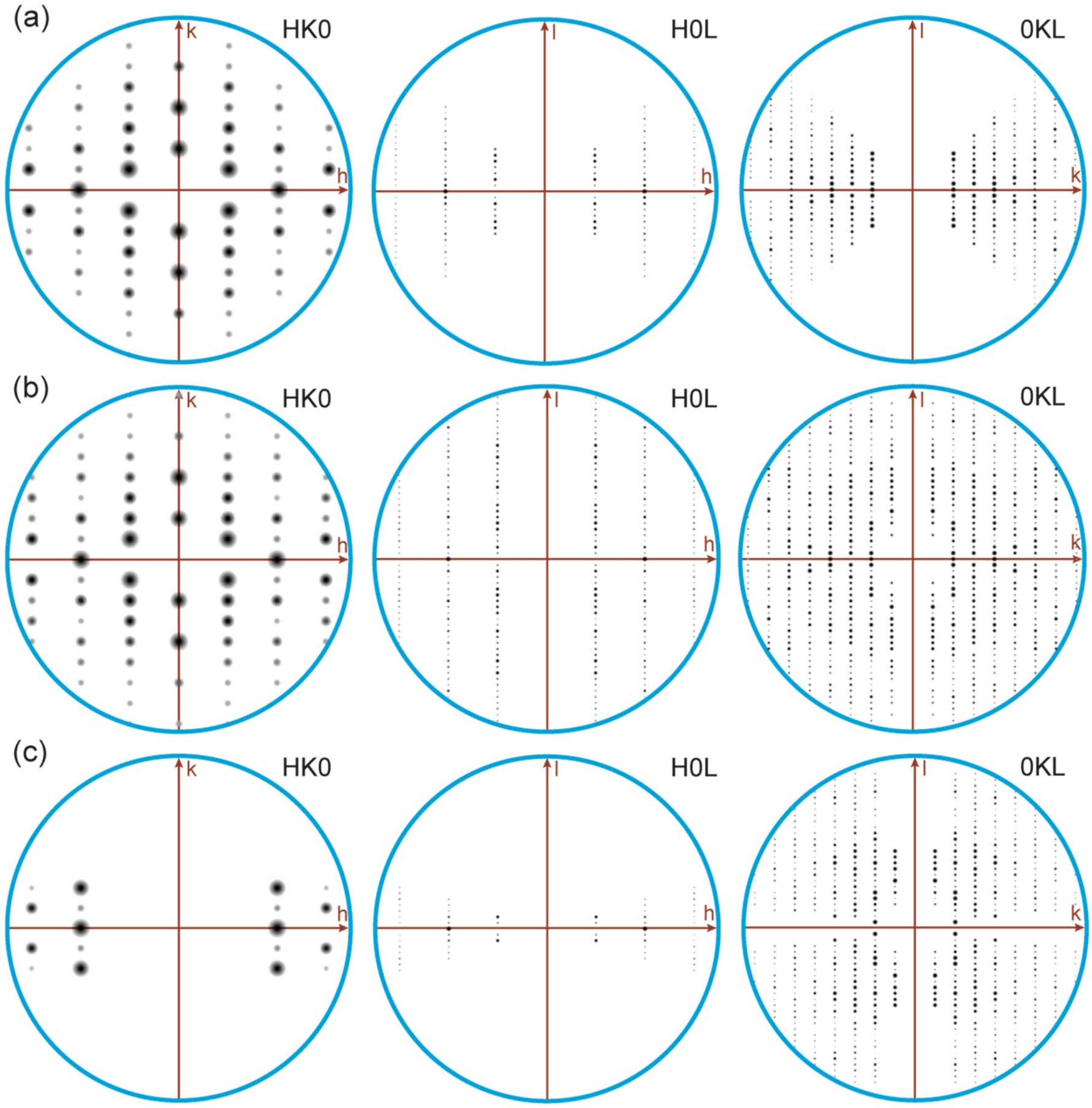

Figure S9 Comparison of Integrated intensities at principle zone axes. (*a*) nanoEDT. (*b*) MicroED dataset. (*c*) fixed-angle SAED dataset.

Table S1 Comparison of crystallographic R factors between merged and unmerged microED data.

**Dose (e^-/Å^2)**	**Single crystal (R_{work}/R_{free})**	**3 crystal merge (R_{work}/R_{free})**
3	23/24	20/24
6	25/28	23/24
9	27/38	25/25
12	Could not refine	27/36
